# Interstitial hydrogen atoms in face-centered cubic iron in the Earth’s core

**DOI:** 10.1038/s41598-019-43601-z

**Published:** 2019-05-08

**Authors:** Daijo Ikuta, Eiji Ohtani, Asami Sano-Furukawa, Yuki Shibazaki, Hidenori Terasaki, Liang Yuan, Takanori Hattori

**Affiliations:** 1 Department of Earth Science, Tohoku University, Sendai, Miyagi 980-8578 Japan; 2 Materials and Life Science Division, Japan Proton Accelerator Research Complex (J-PARC) Center, Japan Atomic Energy Agency, Naka, Ibaraki 319-1195 Japan; 3 Frontier Research Institute for Interdisciplinary Sciences, Tohoku University, Sendai, Miyagi 980-8578 Japan; 4 Department of Earth and Space Science, Osaka University, Toyonaka, Osaka 560-0043 Japan; 5 Present Address: National Institute for Materials Science, Tsukuba, Ibaraki 305-0044 Japan

**Keywords:** Geochemistry, Core processes

## Abstract

Hydrogen is likely one of the light elements in the Earth’s core. Despite its importance, no direct observation has been made of hydrogen in an iron lattice at high pressure. We made the first direct determination of site occupancy and volume of interstitial hydrogen in a face-centered cubic (fcc) iron lattice up to 12 GPa and 1200 K using the *in situ* neutron diffraction method. The transition temperatures from the body-centered cubic and the double-hexagonal close-packed phases to the fcc phase were higher than reported previously. At pressures <5 GPa, the hydrogen content in the fcc iron hydride lattice (*x*) was small at *x* < 0.3, but increased to *x* > 0.8 with increasing pressure. Hydrogen atoms occupy both octahedral (O) and tetrahedral (T) sites; typically 0.870(±0.047) in O-sites and 0.057(±0.035) in T-sites at 12 GPa and 1200 K. The fcc lattice expanded approximately linearly at a rate of 2.22(±0.36) Å^3^ per hydrogen atom, which is higher than previously estimated (1.9 Å^3^/H). The lattice expansion by hydrogen dissolution was negligibly dependent on pressure. The large lattice expansion by interstitial hydrogen reduced the estimated hydrogen content in the Earth’s core that accounted for the density deficit of the core. The revised analyses indicate that whole core may contain hydrogen of 80(±31) times of the ocean mass with 79(±30) and 0.8(±0.3) ocean mass for the outer and inner cores, respectively.

## Introduction

The Earth’s core has supposed to be constituted by iron with ~10% nickel and some light elements. Hydrogen is one of the most probable candidates among the light elements in the Earth’s core. In order to estimate the hydrogen contents in the inner and outer cores, previous studies have determined phase relations, equations of state, magnetic properties, and the sound velocity of iron hydride FeH_*x*_ by using synchrotron X-ray^1–7^ and theoretical works^8^. Despite intensive studies of iron hydride by X-ray diffraction, precise *in situ* direct determination of the volume expansion by interstitial hydrogen in metallic iron has not yet been conducted using *in situ* high pressure and high temperature neutron diffraction studies, except the neutron diffraction of recovered iron hydrides at 90 K and ambient pressure^9^. Therefore, we need to use the volume of hydrogen estimated empirically from many metal hydride compounds^10^. To date, neutron diffraction experiments at high pressures have been conducted for deuterides^11,12^, but not for hydrides. However, the experimental results for deuterium (D) compounds cannot be directly applied to discuss the behaviors of hydrogen compounds in the lower mantle and core because physical and thermodynamic properties of deuterium and hydrogen compounds differ with each other.

In this study, we directly determined the crystallographic positions of hydrogen atoms and their effects on cell volumes for a high-pressure polymorph, fcc iron hydride, using neutron powder diffraction measurements. This technique provided a precise determination of the hydrogen content in the samples used in previous studies on the phase relations and equation of state of iron hydride. Thus, we can estimate precisely the hydrogen content in the core by comparison with the seismic model of the Earth’s core.

## Results

**Figure 1:**
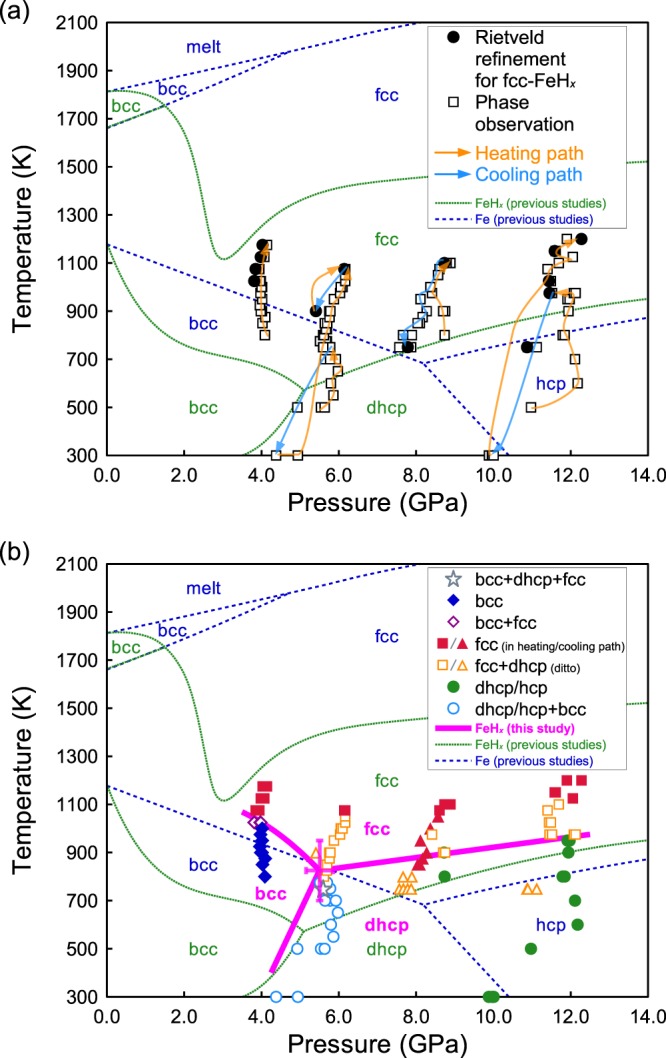
The experimental pressure and temperature conditions and observed phase(s) of FeH_*x*_ in this study. (**a**) The solid circles and open squares represent the experimental conditions of Rietveld refinement for fcc FeH_*x*_ and phase observation, respectively. The yellow and blue arrows indicate the heating and cooling paths, respectively. (**b**) The observed phase(s) in all experimental conditions. The uncertainties in temperature are estimated to be ±50 K. The overall uncertainties in pressure are estimated to be <0.5 GPa; this includes the differences in pressure of 0.2–0.3 GPa before and after neutron diffraction measurement and experimental errors of ~0.1 GPa. Colored symbols show the observed phase(s). Gray star: bcc + dhcp + fcc, blue solid diamonds: bcc, magenta open diamonds: bcc + fcc, red solid squares/triangles: fcc (in heating/cooling paths, respectively), yellow open squares/triangles: fcc + dhcp (in heating/cooling paths, respectively), green solid circles: dhcp/hcp, light blue open circles: dhcp/hcp + bcc. The hcp phase was observed under low temperature conditions where no hydrogen was released from the hydrogen source, NH_3_BH_3_. The magenta bold line represents the phase boundary of FeH_*x*_ phases obtained in this study. The green dotted line and the blue dashed line represent the phase boundaries of FeH_*x*_ and Fe as reproduced from previous studies, respectively^15,16,26^.

**Figure 2:**
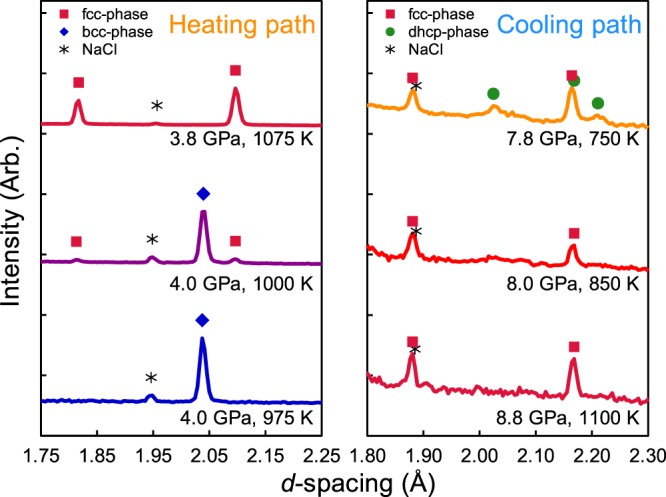
Typical examples of the phase transition for FeH_*x*_ in the heating and the cooling paths. Colored symbols indicate the characteristic peaks of each observed phase (red squares: fcc FeH_*x*_, green circles: dhcp FeH_*x*_, blue diamonds: bcc Fe, black asterisks: NaCl). The left column shows a typical example of the phase transition in the heating path from a single bcc Fe phase through the two phases of bcc Fe and fcc FeH_*x*_, and to single fcc FeH_*x*_. The single bcc Fe phase was observed up to 4.0 GPa, 975 K. The fcc FeH_*x*_ phase appeared at 4.0 GPa, 1000 K with bcc Fe phase, and the bcc Fe disappeared at 3.8 GPa, 1075 K. The right column shows a typical example of phase transition in the cooling path from the single fcc FeH_*x*_ phase to the two phases of fcc FeH_*x*_ and dhcp FeH_*x*_. The single fcc FeH_*x*_ phase at 8.8 GPa, 1100 K was gradually cooled to 750 K, as shown in Fig. 1. The single fcc FeH_*x*_ phase was observed up to 8.0 GPa, 850 K and dhcp FeH_*x*_ clearly appeared at 7.8 GPa, 750 K. The neutron diffraction patterns for phase observation were obtained in each *P*-*T* condition by 5–10 min keeping time and 5–10 min exposure time after increasing or decreasing the temperature.

We determined the volume expansion by interstitial hydrogen atoms in the fcc iron hydride lattice by neutron diffraction at various pressures and temperatures for the first time. The phase transition and hydrogenation of iron were observed in both the fcc FeH_*x*_ and the double-hexagonal close-packed (dhcp) FeH_*x*_ phases. We conducted four separate runs from 3.5 to 12 GPa and in the temperature range from the ambient to 1200 K by using the cubic press (ATSUHIME)^13^ installed at high-pressure neutron beamline PLANET^14^ in J-PARC. The phase diagram determined by the present *in situ* neutron diffraction experiments and the pressure-temperature paths of our experiments are shown in Fig. 1. The phase boundaries of FeH_*x*_ compounds determined in this study were significantly different from those estimated previously by using electrical resistivity change due to the phase transitions^15,16^; that is, the transition temperature from body-centered cubic (bcc) to fcc was higher than that previously reported in the Fe-H system (Fig. 1). This discrepancy in the phase boundary might be caused by the reaction kinetics of the fcc-bcc-dhcp reactions in FeH_*x*_. We conducted time studies at the conditions of the phase boundaries (Fig. 2). We also observed through the neutron diffraction patterns the phase transitions of forward and reverse reactions *in situ* by increasing and decreasing the temperature at a constant press load condition (Figs 1 and 2). Therefore, our phase boundaries determined by forward and reverse reactions were more reliable than those reported previously which were obtained indirectly by one-way reaction.

The fcc iron lattice has two interstitial sites available for accommodating hydrogen atoms, one octahedral (O) and one tetrahedral (T). In transition metals with an fcc lattice, dissolved hydrogen atoms preferentially occupy the O-site with a free space larger than that of the T-site^17^. T-site occupation has been reported for fcc PdD_*x*_^18^ and FeD_*x*_^11^.

**Figure 3:**
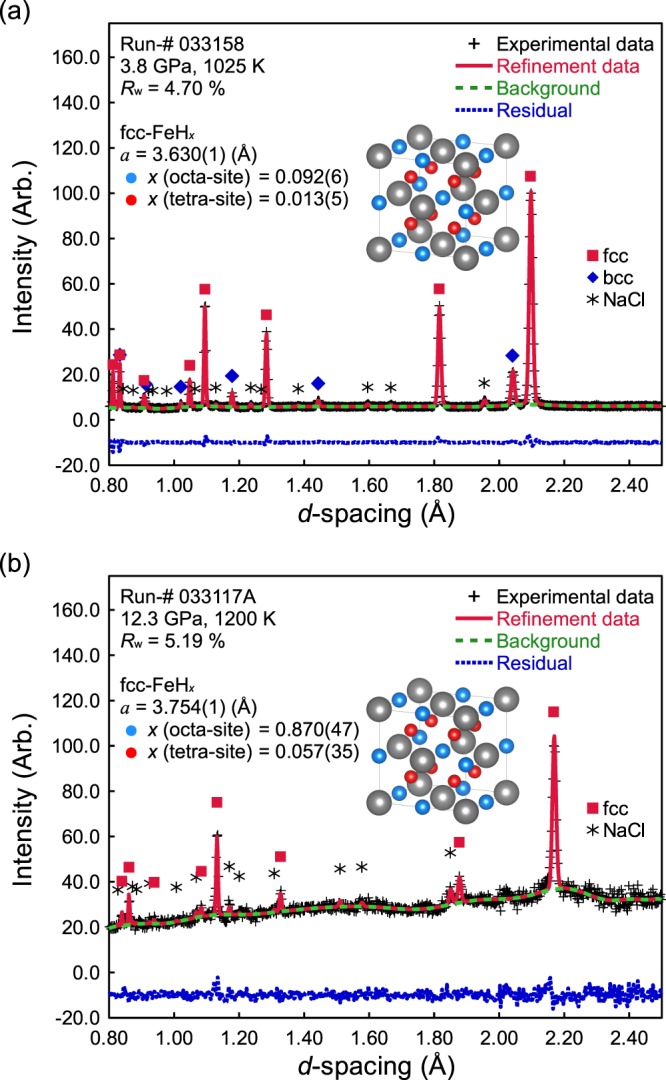
Typical experimental profiles and fitting results of Rietveld refinement for fcc FeH_*x*_. The black + signs represent the neutron diffraction profile obtained experimentally. The red solid line represents refined profiles calculated from Rietveld refinement for fcc FeH_*x*_ (and bcc Fe) with NaCl capsule. The green dashed line represents the background of neutron diffraction profile obtained experimentally. The blue dotted line represents the differences between experimental and refined profiles by an offset of −10 in the vertical axis for clarity. Colored symbols indicate the characteristic peaks of each observed phase (red squares: fcc FeH_*x*_, blue diamonds: bcc Fe, black asterisks: NaCl). The inset figure shows the crystal structure of fcc FeH_*x*_ (the crystal symmetry is cubic (Fm-3m, Z = 4)) and the atomic sites of Fe are 4*a* (0, 0, 0), O-sites of fcc FeH_*x*_ are 4*b* (1/2, 1/2, 1/2), and T-sites of fcc FeH_*x*_ are 8*c* (1/4, 1/4, 1/4). Gray balls represent the Fe atom, light blue balls represent an O-site, and light red balls represent a T-site. The experimental conditions and fitting parameters of Rietveld refinement are given in the figures.

This is the first report for site occupancy of hydrogen in both O- and T-sites in the fcc iron hydride lattice. Figure 3 shows examples of a powder neutron diffraction pattern taken at 3.8 GPa, 1025 K and 12.3 GPa, 1200 K with those Rietveld refinements. To estimate the hydrogen content precisely, we performed Rietveld refinement by three different structural models of the fcc iron lattice reported by Machida *et al*.^11^: (A) the fcc iron lattice without hydrogen, (B) the fcc iron lattice with hydrogen in only O-sites and (C) the fcc iron lattice with hydrogen in both O- and T-sites. Better refinements have been obtained by using the hydride models B and C instead of the non-hydride model A in all powder neutron diffraction patterns. Especially in some powder neutron diffraction patterns, the best refinements are obtained by the hydride model C. As one of the examples for best refinement, the weighted reliability factor of Rietveld refinement of the non-hydride model A, and the hydride models B and C are 7.42%, 5.71%, and 4.70%, respectively and reduced chi-square are 1.47, 1,31, and 1.23, respectively at 3.8 GPa, 1025 K as shown in Supplementary Fig. S1. Although high background considered as contribution by incoherent scattering from hydrogen was observed in large contents of the hydrogen as shown in Fig. 3b, the intensities were sufficient to conduct Rietveld refinement. The results of the Rietveld refinement for fcc FeH_*x*_ in the present experiments are given in Supplementary Table 1. The present analysis showed that hydrogen atoms enter both O- and T-sites. The site occupancy of the hydrogen atoms is also given in Supplementary Table 1. The amounts of hydrogen in T-sites depend on pressure and temperature. Hydrogen atoms occupy both O- and T-sites >1025 K; however, below that temperature, diffraction patterns can be fitted by O-site occupancy for all hydrogen atoms without T-sites. At pressures <5 GPa, the hydrogen content is relatively low at *x* < 0.3, and it increases to *x* > 0.3 at higher pressure. The site occupancy is 0.870(±0.047) for O-sites and 0.057(±0.035) for T-sites at 12 GPa and 1200 K, and at 4 GPa and 1175 K, the occupancy is 0.140(±0.009) for O-sites and 0.018(±0.005) for T-sites. The site occupancies of hydrogen in O- and T-sites are similar to those reported by Machida *et al*.^11^ for deuterium: 0.532(±0.009) for O-sites and 0.056(±0.005) in T-sites, giving a deuterium content (*x*) of 0.64(±0.01) at 6.3 GPa and 988 K.

Hydrogen atoms generally occupy interstitial O-sites of the iron lattice with a small number of T-sites, therefore the iron lattice expands by hydrogenation. The volume expansion of the iron lattice can be calculated from following formula:

$${\Delta }V({\rm{H}})=[V({{\rm{F}}{\rm{e}}{\rm{H}}}_{x})-V({\rm{F}}{\rm{e}})]/x,$$

where *x*, *V*(FeH_*x*_), *V*(Fe), and *ΔV*(H) are the hydrogen concentration, atomic volumes of iron hydride and pure iron, and the volume expansion per hydrogen atom, respectively^19^. We used the equation of state of fcc iron reported by Tsujino *et al*.^20^ for *V*(Fe). Here, *V*(FeH_*x*_) and the hydrogen concentration (*x*) at each pressure and temperature were directly determined by powder neutron diffraction combined with Rietveld refinement, and we could determine the volume expansion per hydrogen atom, *ΔV*(H), in this study. To date, there have been a limited number of experiments on the determination of *ΔV*(H) in the fcc lattice of metals, such as the neutron diffraction study of the quenched iron-rich hydride alloy, Fe_0.65_Mn_0.29_Ni_0.06_H_0.95_, synthesized by Antonov *et al*.^21^ at 7 GPa, and recovered to atmospheric pressure at liquid N_2_ temperature. They determined the hydrogen concentration by degassing after recovery. The *ΔV*(H) value of 1.9 Å^3^ that they obtained is consistent with that of other 3d transition-metal hydrides with fcc crystal structure (e.g., γ-MnD_0.45_: 1.85 Å^3^, γ-CoH: 1.9 Å^3^)^17^. The present measurement of a volume increase by a hydrogen atom, *ΔV*(H), can be compared with this value and is discussed later.

**Figure 4:**
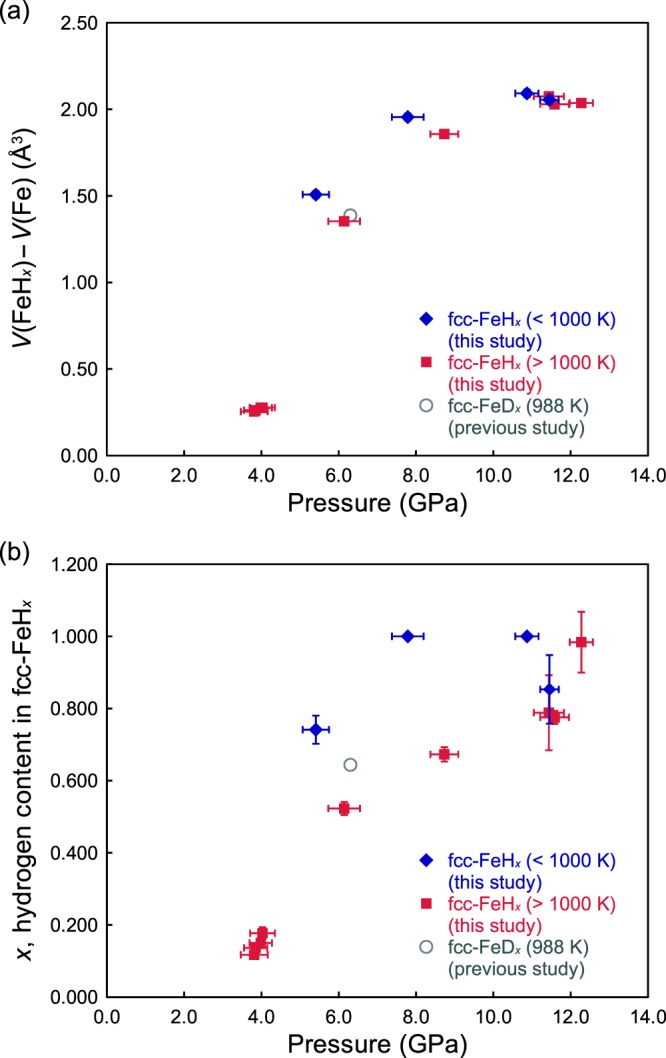
(**a**) Pressure dependence of the volume expansion [=*V*(FeH_*x*_) − *V*(Fe)] from fcc Fe to fcc FeH_*x*_ due to hydrogen dissolution. (**b**) Pressure dependence of the hydrogen content *x* in fcc FeH_*x*_. The blue diamonds represent the temperature conditions <1000 K and the red squares represent the temperature conditions >1000 K. A gray circle represents the result of iron deuteride (FeD_*x*_) at 6.3 GPa and 988 K^11^. The errors in pressure are defined in Fig. 1. Other error bars represent the 1*σ* uncertainty.

The pressure dependence of the volume increase in the fcc iron lattice by hydrogen dissolution, *V*(FeH_*x*_) − *V*(Fe), and its hydrogen content, *x*, are shown in Fig. 4. As can be observed, both *V*(FeH_*x*_) − *V*(Fe) and *x* increase with increasing pressure; *V*(FeH_*x*_) − *V*(Fe) depends very weakly on temperature, whereas *x* decreases with increasing temperature.

**Figure 5:**
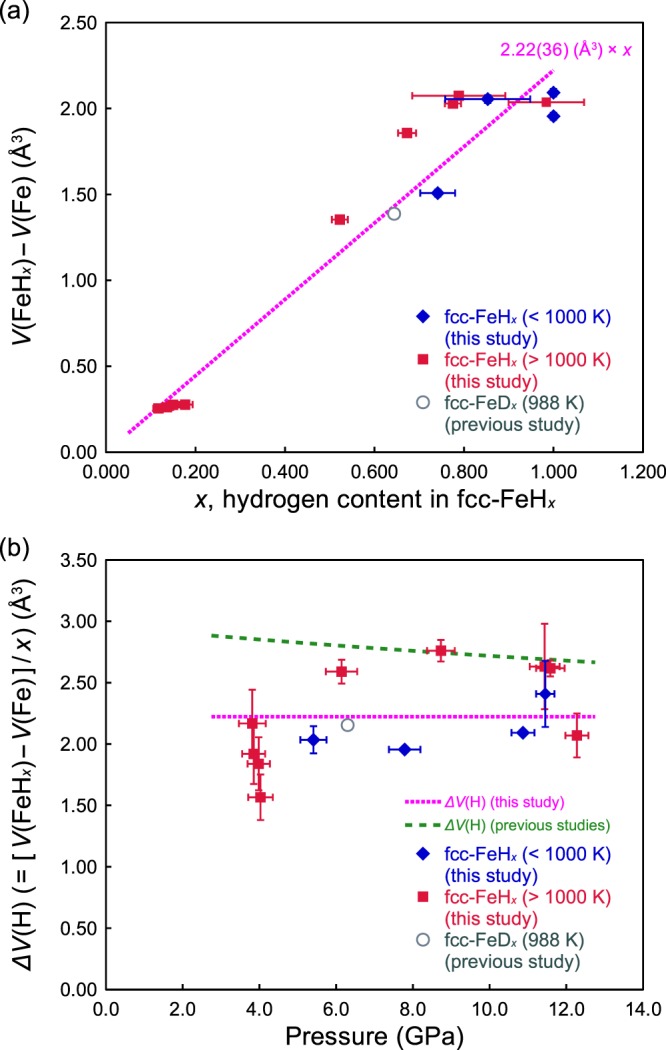
(**a**) Compositional dependence of the volume expansion of the Fe lattice per Fe atom [=*V*(FeH_*x*_) − *V*(Fe)] due to hydrogen dissolution. The volume expansion due to dissolution of one hydrogen atom *ΔV*(H) {=[*V*(FeH_*x*_) − *V*(Fe)]/*x*} is 2.22(±0.36) Å^3^ shown as a magenta dotted line. (**b**) Pressure dependence of the volume expansion of the fcc lattice due to dissolution of one hydrogen atom, *ΔV*(H). The pressure dependence calculated theoretically^4,11^ is shown as a green dashed line. The abbreviations of symbols and the errors are the same as those in Fig. 4.

Figure 5a shows the magnitude of the volume expansion *V*(FeH_*x*_) − *V*(Fe) for the fcc iron lattice increases with increasing the hydrogen content, *x*. The volume expansion *V*(FeH_*x*_) − *V*(Fe) of the lattice seems to increase linearly up to *x* ~0.8, at a rate of *ΔV*(H) of ~2.22(±0.36) Å^3^ at high temperatures, which is comparable with that of deuterium (*ΔV*(D) ~2.21 Å^3^) determined at 988 K and 6.3 GPa by Machida *et al*.^11^. Figure 5b shows the pressure dependence of the volume increase of the fcc lattice as a result of hydrogen dissolution for one hydrogen atom, *ΔV*(H). The figure indicates that *ΔV*(H) is almost independent of pressure at least up to 12 GPa.

## Discussion

Several high pressure polymorphs of iron hydride and superhydride phases were reported by using *in situ* X-ray diffraction method. Hirao *et al*.^5^ observed dhcp FeH_*x*_ phases up to 80 GPa and 300 K. Peppin *et al*.^6,7^ observed iron hydride, FeH_2_ and FeH_3_ at around 60–80 GPa, and iron superhydride phase, FeH_5_ toward to 130–160 GPa after temperature quenching at room temperature. In theoretical work, the presence of further superhydride phase FeH_6_ has been suggested^8^. However, these studies were conducted only at room temperature conditions, and had not observed the behavior of hydrogen and its volume *in situ*. Therefore, our data provide important information for evaluation of the effect of hydrogen dissolution on the compression behavior of the fcc iron lattice that is a high pressure and high temperature phase of FeH_*x*_, and for reliable estimation of the hydrogen content in the Earth’s core by comparison with the density of the PREM (Preliminary Reference Earth Model) Earth’s core^22^. Chakravarty *et al*.^23^ made *ab initio* calculations for hydrogen in the iron lattice and showed the pressure effect on the volume of interstitial hydrogen at high pressure. The interstitial volume *ΔV*(H)^23^ fitted using the Vinet equation of state by Fukai^19^ (Fig. 5b) was very weakly dependent on pressure, which is consistent with the present work.

The volume increase per hydrogen atom, *ΔV*(H), in fcc FeH_*x*_ has not been determined previously, and the value of *ΔV*(H) = 1.9 Å^3^ per hydrogen atom that is conventionally used for fcc FeH_*x*_ was estimated by the neutron diffraction of fcc (Fe_0.65_Mn_0.29_Ni_0.06_)H_0.95_ cooled to ~220 K and recovered under ambient pressure^21^. Sakamaki *et al*.^1^ used this value of volume expansion and reported that fcc FeH_*x*_ approaches superstoichiometric composition with *x* = 1.0–1.2 immediately below the melting temperature at 11.5–20 GPa. The present results of lattice expansion per hydrogen atom, *ΔV*(H) = 2.22(±0.36) Å^3^, at high temperature modify the above arguments; that is, the hydrogen content in fcc FeH_*x*_ in the experiments reported by Sakamaki *et al*.^1^ should be *x* = 0.86–1.03, which is not superstoichiometric but is close to the stoichiometric composition, FeH.

Narygina *et al*.^2^ synthesized fcc FeH_*x*_ at 30 GPa and >1600 K and determined the equation of state of this phase. Based on its compression curve, they estimated the hydrogen content of their synthesized sample to be *x* = 0.9–1.3 using the volume expansion per hydrogen atom, *ΔV*(H) = 1.9 Å^3^, and concluded that the amount of hydrogen required to match the density of the Earth’s core would be 0.5–1 wt.% in the outer core and 0.08–0.16 wt.% in the inner core. Thompson *et al*.^3^ also studied the hydrogen content in the core based on the equation of state and nuclear resonant inelastic X-ray scattering studies of fcc FeH_*x*_; by using the conventional *ΔV*(H) value of 1.9 Å^3^, they estimated that the outer core contains 0.8–1.1 wt.% hydrogen, whereas the inner core contains 0.2–0.3 wt.% hydrogen. The hydrogen content in the core estimated in these studies^2,3^, that is, 0.5–1.1 wt.% for the outer core and 0.08–0.2 wt.% for the inner core, was overestimated and can be recalculated to 0.65(±0.25) wt.% in the outer core and 0.12(±0.05) wt.% in the inner core by using *ΔV*(H) = 2.22(±0.36) Å^3^ determined in this study. The revised analyses indicate that whole core may contain hydrogen of 80(±31) times of the ocean mass with 79(±30) and 0.8(±0.3) ocean mass for the outer and inner cores, respectively.

## Methods

The high-pressure and high-temperature neutron diffraction experiments were conducted at the high-pressure PLANET beamline (BL11) at the Material and Life Science Experimental Facility (MLF) in J-PARC^14^. The PLANET beamline has the capability to obtain low background including incoherent scattering from hydrogen due to the excellent collimation of the incident beam and scattered neutron with narrow incident and receiving collimators^14^. A six-axis multi-anvil high-pressure apparatus (ATSUHIME)^13^ installed at this beamline was used for high-pressure generation. An iron disc specimen was placed in the center of a hydrogen-sealing capsule made of NaCl with internal hydrogen sources of NH_3_BH_3_ pellets above and below^24^. To seal hydrogen, the NaCl capsule was inserted in a cylindrical graphite heater^1,16^ and embedded in a pressure-transmitting medium made of Cr-doped MgO [a 10.5 mm edge cube for the anvil with a 7 mm truncated edge length (TEL), and a 15 mm edge cube for the anvil with a 10 mm TEL]. Neutron diffraction data were collected at high pressure and high temperature. The generated pressure was determined by the neutron powder diffraction profile of the capsule material of NaCl. The NaCl-B1 pressure scale used in this experiment was based on Brown^25^. The experimental temperature was evaluated based on the heating power using a power-temperature calibration curve which was determined by the separate runs with a Pt-Pt 13% Rh thermocouple^11^. The accuracy of the temperature calibration curve was confirmed by the bcc-fcc transition of iron^26^. The uncertainty of the temperature determination was ± 50 K. Neutron diffraction profiles were collected during increasing and decreasing temperatures for 5 min. The temporal evolution of the diffraction profile was monitored at several fixed temperatures near the phase boundaries above the decomposition temperature of NH_3_BH_3_^24^ which is considered to be <500 K as examples shown in Fig. 2. In addition, no unknown phases were observed except Fe, FeH_*x*_, and NaCl in both heating/cooling paths (Figs 2 and 3). This indicates that the reaction had been occurred only by iron and hydrogen, and there was no contamination of iron hydride sample by boron or nitrogen from the hydrogen sources. The composition and site occupancy of hydrogen atoms in the fcc lattice of FeH_*x*_ were determined for the equilibrium state of fcc FeH_*x*_ at a fixed pressure based on Rietveld analysis of the diffraction profiles. The temperature was kept constant and the temporal evolution of the diffraction profile was monitored to confirm that FeH_*x*_ reached equilibrium with the surrounding H_2_ fluid. High-pressure and high-temperature neutron diffraction experiments were conducted at pressures in the range 3–12 GPa and at temperatures in the range 900–1200 K. Two runs (Runs 1 and 4) were conducted in the pressure range 3.5–6 GPa using cubic anvils with a 10 mm truncated edge. The other two runs (Runs 2 and 3) were conducted at pressures in the range 8–12 GPa using anvils with a 7 mm truncated edge. The schematic diagram of the multianvil apparatus with the high-pressure and high-temperature cell assembly and the neutron scattering geometry is given in Machida *et al*.^11^. The diffraction intensity of the sample was corrected using the data obtained for a vanadium pellet and an empty cell of a dimension similar to that used in sample data collection. The detailed cell assembly of the present cubic apparatus is given in Supplementary Fig. 2.

## Supplementary information

Supplementary Information

## Data Availability

All data supporting the findings of this study are available within the paper, Methods and Supplementary Information. The crystallographic data are available from the corresponding authors upon request.
