# Supplementary material for: Interstitial hydrogen atoms in face-centered cubic iron in the Earth’s core
Source: Sci Rep. 2019 May 8;9:7108. doi: 10.1038/s41598-019-43601-z (PMC6506596; doi:10.1038/s41598-019-43601-z)
Supplement: Supplementary file 1 — Supplementary Information [file 41598_2019_43601_MOESM1_ESM.pdf]

Supplementary Information for

Interstitial hydrogen atoms in face-centered cubic iron in the Earth's core

Daijo Ikuta*, Eiji Ohtani*, Asami Sano-Furukawa, Yuki Shibazaki,
Hidenori Terasaki, Liang Yuan, Takanori Hattori

* Correspondence and requests for materials should be addressed to
D.I. (email: dikuta@tohoku.ac.jp) or E.O. (email: eohtani@tohoku.ac.jp)

This PDF file includes:

Supplementary Figures S1 to S2
Supplementary Table S1
Reference for SI reference citation

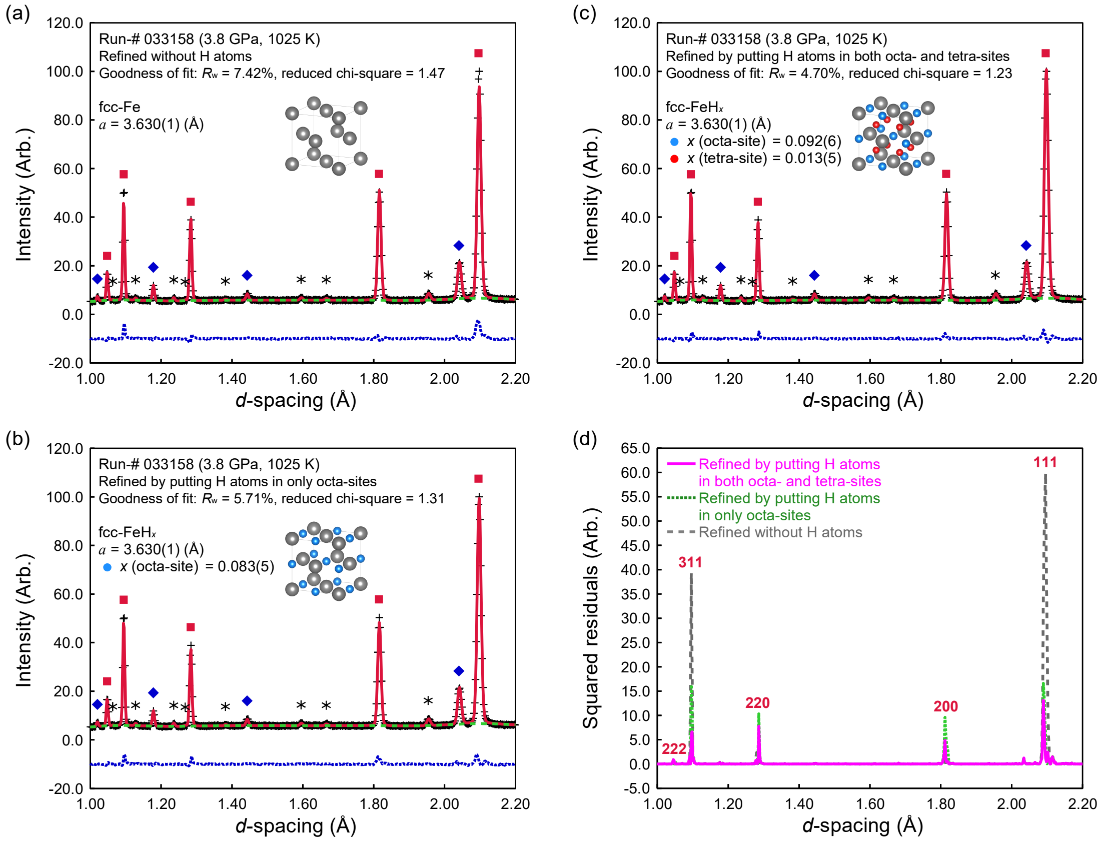

Supplementary Figure S1.

Typical experimental profiles and fitting results of Rietveld refinement for (a) the non-hydride model A: fcc Fe without hydrogen, (b) the hydride model B: fcc FeH_x with hydrogen in only O-sites and (c) the hydride model C: fcc FeH_x with hydrogen in both O- and T-sites. The inset figures represent the applied structural model of fcc iron lattice in Rietveld refinement, respectively. The abbreviations of the symbols and the errors are the same as in Fig. 3. (d) The gray dashed, green dotted, and magenta solid lines represent the squared residuals after Rietveld refinement by the model of A, B, and C, respectively. The red numbers indicate the *hkl* Miller indices for each peak of the fcc iron lattice. The squared residuals of 111 and 311 peaks are significantly decrease by using the hydride model B and C instead of non-hydride model A. The squared residuals of four peaks (111, 200, 220, and 311 peaks) are further decrease by 30-65% by using the hydride model C rather than the hydride model B. Because the residuals of the peaks of fcc iron lattice are obviously improved, the best fitting is obtained by using the hydride model C, fcc FeH_x with hydrogen in both O- and T-sites as shown in Fig. S1(c) than the other models A and B as shown in Fig. S1(a, b).

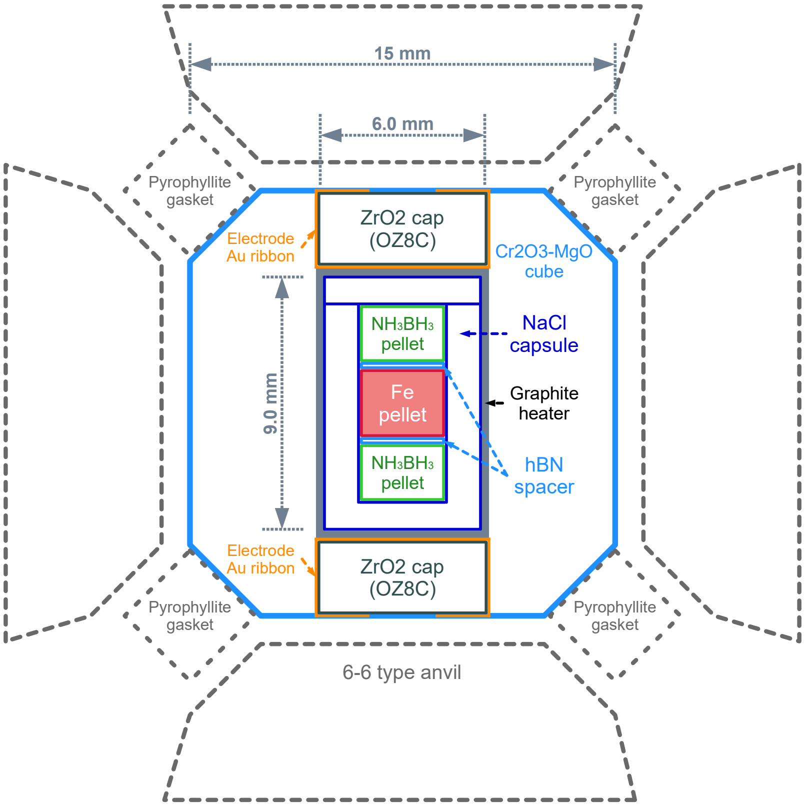

Supplementary Figure S2.

A two-dimensional schematic diagram of a high-pressure and high-temperature cell.

Supplementary Table S1. Results of Rietveld refinement for fcc FeH_x.

	Experimental condition		Rietveld refinement for *fcc*-FeH_x: Unit cell parameter		Site occupancy of hydrogen atom			Atomic displacement parameter				observed	Unit cell volume of *fcc*-Fe (Å^3)	Atomic volume expansion due to hydrogen dissolution	
Run-#	P (GPa)	T (K)	a (Å)	V (Å^3)	x (octa-site)	x (tetra-site)	x (total)	U_{iso} (Fe)	U_{iso} (octa)	U_{iso} (tetra)	R_w (%)	phase(s)	(Tsujino *et al.*, 2013)	$V(FeH_x) - V(Fe)$ (Å^3)	$\Delta V(H)$ (Å^3)
Run1-028487	5.4 (3)	900 (50)	3.733 (1)	52.00 (1)	0.741 (39)	0 (fixed)	0.741 (39)	0.004 (1)	0.029 (7)	-	3.29	*fcc+dhcp*	45.96	1.51 (2)	2.03 (11)
Run1-028490	6.1 (4)	1075 (50)	3.723 (1)	51.60 (1)	0.523 (18)	0 (fixed)	0.523 (18)	0.006 (4)	0.023 (3)	-	4.00	*fcc*	46.18	1.35 (2)	2.59 (10)
Run2-028541	8.7 (4)	1100 (50)	3.754 (1)	52.91 (1)	0.673 (20)	0 (fixed)	0.673 (20)	0.005 (1)	0.027 (2)	-	3.17	*fcc*	45.47	1.86 (2)	2.76 (9)
Run2-028555	7.8 (4)	750 (50)	3.750 (1)	52.74 (1)	1 (fixed)	0 (fixed)	1 (fixed)	0.009 (2)	0.018 (5)	-	5.43	*fcc+dhcp*	44.92	1.96 (2)	1.96 (2)
Run3-033094	11.5 (2)	975 (50)	3.749 (1)	52.69 (4)	0.853 (95)	0 (fixed)	0.853 (95)	0.011 (3)	0.04 (2)	-	2.77	*fcc+dhcp*	44.46	2.05 (2)	2.41 (27)
Run3-033098	10.9 (3)	750 (50)	3.745 (1)	52.50 (4)	1 (fixed)	0 (fixed)	1 (fixed)	0.006 (3)	0.05 (1)	-	3.05	*fcc+dhcp*	44.13	2.09 (2)	2.09 (2)
Run3-033105	11.4 (4)	1050 (50)	3.753 (1)	52.88 (4)	0.788 (104)	0 (fixed)	0.788 (104)	0.010 (3)	0.04 (2)	-	2.54	*fcc+dhcp*	44.58	2.07 (2)	2.63 (35)
Run3-033115	11.6 (4)	1150 (50)	3.755 (1)	52.94 (1)	0.775 (18)	0 (fixed)	0.775 (18)	0.005 (1)	0.024 (2)	-	2.83	*fcc*	44.81	2.03 (2)	2.62 (7)
Run3-033117A	12.3 (3)	1200 (50)	3.754 (1)	52.90 (1)	0.870 (47)	0.057 (35)	0.984 (84)	0.005 (1)	0.023 (4)	0.03 (6)	5.19	*fcc*	44.74	2.04 (2)	2.07 (18)
Run4-033158	3.8 (4)	1050 (50)	3.630 (1)	47.82 (1)	0.092 (6)	0.013 (5)	0.117 (12)	0.006 (2)	0.014 (5)	0.06 (6)	4.70	*bcc+fcc*	46.80	0.25 (2)	2.17 (27)
Run4-033161	3.8 (3)	1075 (50)	3.634 (1)	47.98 (1)	0.112 (7)	0.012 (6)	0.136 (14)	0.007 (2)	0.015 (5)	0.06 (7)	5.49	*fcc*	46.93	0.26 (2)	1.92 (24)
Run4-033163	4.0 (3)	1150 (50)	3.638 (1)	48.13 (1)	0.122 (7)	0.014 (6)	0.150 (14)	0.007 (2)	0.022 (5)	0.07 (6)	5.17	*fcc*	47.02	0.28 (2)	1.84 (22)
Run4-033165	4.0 (3)	1175 (50)	3.641 (1)	48.26 (1)	0.140 (9)	0.018 (7)	0.177 (17)	0.007 (2)	0.026 (6)	0.06 (5)	6.08	*fcc*	47.15	0.28 (2)	1.57 (19)

Abbreviations: P: pressure (GPa), T: temperature (K), a: unit cell parameter of fcc FeH_x (Å), V: unit cell volume of fcc FeH_x (Å^3), x(octa-site): site occupancy of hydrogen atom in octahedral sites [4*b* (1/2, 1/2, 1/2)] for fcc FeH_x, x(tetra-site): site occupancy of hydrogen atom in tetrahedral sites [8*c* (1/4, 1/4, 1/4)] for fcc FeH_x, x(total): hydrogen content for fcc FeH_x [= x(octa-site) + 2x(tetra-site)], R_w: weighted reliability factor of Rietveld refinement for fcc FeH_x (%), unit cell volume of fcc Fe: unit cell volume calculated from the equation of state of fcc Fe (Å^3) (Ref. 1), $V(FeH_x) - V(Fe)$: atomic volume expansion from fcc Fe to fcc FeH_x because of hydrogen dissolution (Å^3), $\Delta V(H)$: volume expansion of fcc lattice due to hydrogen dissolution per hydrogen atom {= [$V(FeH_x) - V(Fe)$] / x(total)} (Å^3).

Note: Values in parentheses represent the error of each parameter; "fixed" in parentheses means the parameter was fixed because it was refined to be quite close to 0 or 1 by Rietveld refinement using free parameters and better refinements were obtained through Rietveld refinement using fixed parameter rather than free parameter.

Supplementary Reference

1. Tsujino *et al.*, (2013) Equation of state of γ-Fe: Reference density for planetary cores. *Earth Planet Sci Lett* 375:244-253.
